# Lipid Accumulation Product Is Predictive of Cardiovascular Hospitalizations among Patients with Stable Ischemic Heart Disease: Long-Term Follow-Up of the LAERTES Study

**DOI:** 10.3390/jcdd11100316

**Published:** 2024-10-10

**Authors:** Konstantinos A. Papathanasiou, Christos Eleftherios Roussos, Stylianos Armylagos, Stylianos L. Rallidis, Loukianos S. Rallidis

**Affiliations:** 1 2nd Department of Cardiology, Medical School, National and Kapodistrian University of Athens, University General Hospital ATTIKON, Rimini 1, Chaidari, 12462 Athens, Greece; 2 Biomedical Sciences, University of Nicosia Medical School, Saint Nicholas 93, Egkomi 2408, Cyprus

**Keywords:** stable ischemic heart disease, lipid accumulation product, abdominal adiposity, cardiovascular hospitalizations, acute coronary syndrome

## Abstract

**(1) Background:** Lipid accumulation product (LAP) is an anthropometric index of abdominal adiposity that has been associated with increased cardiovascular risk. We aimed to explore the association of LAP with cardiovascular hospitalizations and compare its predictive accuracy with other indices such as body mass index (BMI) and waist circumference. **(2) Methods:** LAERTES was a prospective, population-based cohort that recruited consecutive patients with stable ischemic heart disease (SIHD) from two Greek hospitals in Athens. Data from 770 participants (13% women, median age 62 years) with a median follow-up of 4.3 years were analyzed in relation to the occurrence of adverse cardiovascular events mandating hospital admission (non-fatal myocardial infarction [MI], non-fatal ischemic stroke and malignant ventricular arrhythmias). **(3) Results:** A total of 127 (16.5%) of the participants were admitted to cardiology clinics over the follow-up period; 12.4% of them developed MI, 2.6% ventricular arrhythmia and 1.5% ischemic stroke. Patients with cardiovascular hospitalization had higher BMI, larger waist circumference, higher LAP and triglycerides and lower HDL-cholesterol than patients without hospitalization. Upper LAP quartile and hypertension were independent predictors for cardiovascular hospitalization (HR: 2.20, 95% CI: 1.12–4.34, *p* = 0.02 and HR: 1.57, 95% CI: 1.03–2.39, *p* = 0.03, respectively). **(4) Conclusions:** Higher LAP quartiles are predictive of adverse cardiovascular events leading to hospital admission and deserve further evaluation in dedicated studies.

## 1. Introduction

Stable ischemic heart disease (SIHD) is among the most prevalent diseases worldwide and its burden will increase in the coming years, driven by population aging and the global obesity epidemic [1]. Obesity is a well-established cardiovascular risk factor, yet body mass index (BMI), commonly employed to define obesity, leads to heterogeneous results in relation to cardiovascular and all-cause mortality [2,3].

Prevention strategies have mainly focused on traditional cardiovascular risk factors, lifestyle modification and established pharmacotherapies [4,5]. Integrating imaging into preventive cardiology is rigorously investigated at present and epicardial fat has been associated with adverse cardiovascular outcomes [5,6,7]. Continued research is needed to assess the cost-effectiveness of using novel cardiovascular imaging methods on a large scale.

Anthropometric indices of obesity are inexpensive and readily available. Importantly, they have been associated with cardiovascular risk in both middle-aged male [8] and female patients [9], although ethnic and gender differences might apply [10]. Novel insulin resistance indices could be indicative of metabolic syndrome and multivessel coronary atherosclerosis among patients with acute coronary syndrome (ACS) [11].

Lipid accumulation product (LAP) was analyzed several years ago in the third National Health and Nutrition Examination Survey (NHANES III) and it is based on waist circumference and fasting serum triglyceride levels [12]. Previous studies have shown that LAP is predictive of increased cardiovascular risk among adults without cardiovascular disease [12,13,14,15], which could be explained by its strong and positive association with metabolic syndrome [16], diabetes mellitus (DM) and hypertension [17]. Recently, the Korean Genome and Epidemiology Study showed that LAP levels had a J-shaped association with all-cause mortality [18].

Data regarding the impact of LAP on long-term adverse cardiovascular events leading to hospital admission among patients with SIHD are scarce. The purpose of this analysis was to explore the aforementioned association.

## 2. Materials and Methods

### 2.1. Study Population

This study is a retrospective analysis from LAERTES (Lipoprotein-Associated phospholipasE A2 in stable coronary aRTEry diSease Study). The study design, data collection and definitions for coronary artery disease (CAD) have been previously described [19]. The following definitions for risk factors were applied: hypertension: blood pressure ≥140/90 mmHg and/or antihypertensive treatment; hypercholesterolemia: low-density lipoprotein cholesterol (LDL-C) >130 mg/dL (3.4 mmol/L) [if on lipid-lowering therapy (LLT), the pretreatment cholesterol levels were calculated by using correction factors described by Besseling et al. [20]]; DM: fasting plasma glucose >125 mg/dL (6.94 mmol/L) and/or glucose-lowering treatment and family history of premature CAD, and the documentation of sudden death or CAD in first-degree male relatives <55 years of age or in first-degree female relatives <65 years of age.

In brief, patients with SIHD attending the outpatient cardiology department of two large hospitals in Athens, Greece (University General Hospital Attikon and General Hospital of Nikea), were consecutively enrolled between 2009 and 2014. Patients were included if they had past history of acute coronary syndrome, had undergone coronary revascularization (percutaneous coronary intervention or coronary artery bypass grafting) or had undergone coronary angiography for chest pain evaluation with subsequent documentation of significant CAD. Subjects with recent (within six months) ACS, heart failure, chronic kidney disease (creatinine levels > 2 mg/dL), active malignancy or inflammatory disease and advanced age (>80 years) were excluded. All patients underwent an echocardiographic study and the ejection fraction of left ventricle (LVEF) was estimated using the biplane method of discs (modified Simpson’s rule).

### 2.2. Biochemical Measurements

Fasting blood samples were collected at the initial appointment and serum levels of total cholesterol, high-density lipoprotein cholesterol (HDL-C) and triglycerides were measured enzymatically on automatic analyzers (Dimension RXL; Dade Behring, Marburg, Germany). LDL-C was calculated according to Friedewald’s formula (LDL-C (mg/dL) = Total cholesterol − HDL-C − Triglycerides/5). Lipoprotein(a) [Lp(a)] was assayed by high-sensitivity particle-enhanced immunonephelometry on the BN ProSpec system (Siemens, Erlagen, Germany). The immunoassay used to quantify Lp(a) was an isoform-insensitive assay. Serum levels of apolipoprotein B (apo B) were measured by immunonephelometry using a BN Prospect analyzer (Dade Behring, Deerfield, IL, USA).

### 2.3. Lipid Accumulation Product at Baseline

LAP, based on baseline waist circumference (cm) and fasting serum triglycerides (mmol/L), was computed in male and female participants with the following equations [12]:

LAP males: (waist circumference-65) × Triglycerides

LAP females: (waist circumference-58) × Triglycerides

In order to convert triglyceride values from mg/dL to mmol/L, we divided the value in mg/dL by 88.57 and LAP quartiles were extracted accordingly.

### 2.4. Follow-Up and Clinical Endpoints

After initial evaluation, all patients were subject to a follow-up program which involved telephone interviews or clinic visits every 6 months. The primary endpoint of this analysis was cardiovascular hospitalizations, which were defined as the composite of non-fatal myocardial infarction (MI), non-fatal ischemic stroke and malignant ventricular arrhythmias.

This study was conducted in accordance with the Declaration of Helsinki, and the protocol was approved by the Ethics Committee of University General Hospital Attikon (552) on 18 March 2011. Informed consent was obtained from all individuals.

### 2.5. Statistical Analysis

Continuous variables are presented as means ± standard deviation (SD) if normally distributed or as medians and interquartile ranges if non-normally distributed. The Kolmogorov–Smirnov test was used to test for normality. Categorical variables are presented as relative frequencies. Comparisons between groups were made using the chi-square test for categorical variables, and Student’s *t* test for independent samples for normally distributed continuous variables or the Mann–Whitney U test for skewed continuous variables. Bivariate correlations were performed using Pearson or Spearman correlation coefficients based on the distribution of the variables under examination. Cox proportional hazards models were used to estimate the hazard ratios (HRs) and the corresponding 95% confidence intervals (CIs) of experiencing a cardiovascular hospitalization during the study follow-up according to the baseline characteristics of the patients. Moreover, receiver operating characteristic (ROC) curve analysis was performed and the corresponding Area Under the Curve (AUC; 95% CI) was also calculated to evaluate the additive value of LAP on top of other known adiposity indices (namely, BMI and waist circumference). A *p*-value < 0.05 was considered statistically significant. IBM SPSS Statistics for Windows, version 28 (IBM Corp., Armonk, NY, USA), was used.

## 3. Results

A total of 770 patients with SIHD were followed for a median follow-up of 52 months (34–72). Cardiovascular hospitalizations occurred in 127 patients (16.5%); 12.4% due to ACS, 2.6% due to ventricular arrhythmias and 1.5% due to ischemic stroke. The patients’ baseline characteristics according to the occurrence of cardiovascular hospitalization are shown in Table 1. The median age was 62 years and females were 13% of the study participants. Smoking and hypertension were the most prevalent cardiovascular risk factors and 80% of the patients had been previously hospitalized for ACS. Patients with cardiovascular hospitalization more commonly suffered from hypertension and obesity. They also exhibited higher BMI and larger waist circumference as compared to patients without cardiovascular hospitalization. Triglycerides and LAP were statistically higher in patients with cardiovascular hospitalization compared to patients without hospital admission, while HDL-C was lower. LAP demonstrated statistically significant correlations with blood pressure and glycated hemoglobin A1 and apoB (Supplementary Table S1 and Supplementary Figure S1).

LVEF and coronary anatomy did not differ between patients with and without the primary clinical endpoint. As far as medical management is concerned, the prescription of antiplatelets, statins, beta-blockers and angiotensin-converting enzyme inhibitors (ACEis) was comparable between the two groups at baseline and during follow-up, except for ACEis, which were more commonly prescribed in patients with cardiovascular hospitalization during the follow-up period. Although the majority of the patients continued to receive LLT (91%) over the follow-up period, less than 20% achieved an LDL-C value <70 mg/dL. Lastly, one in five patients continued to smoke and there was no difference between the two groups. Follow-up parameters and outcomes are summarized in Table 2.

**Figure 1:**
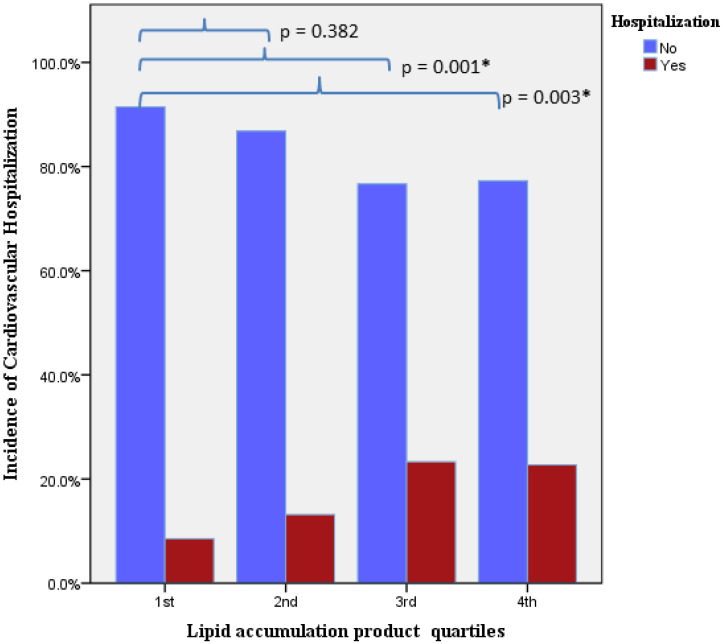
Incidence of cardiovascular hospitalization per lipid accumulation product quartile (LAP) (*p*-value for LAP quartile = 0.002). * statistically significant difference.

**Figure 2:**
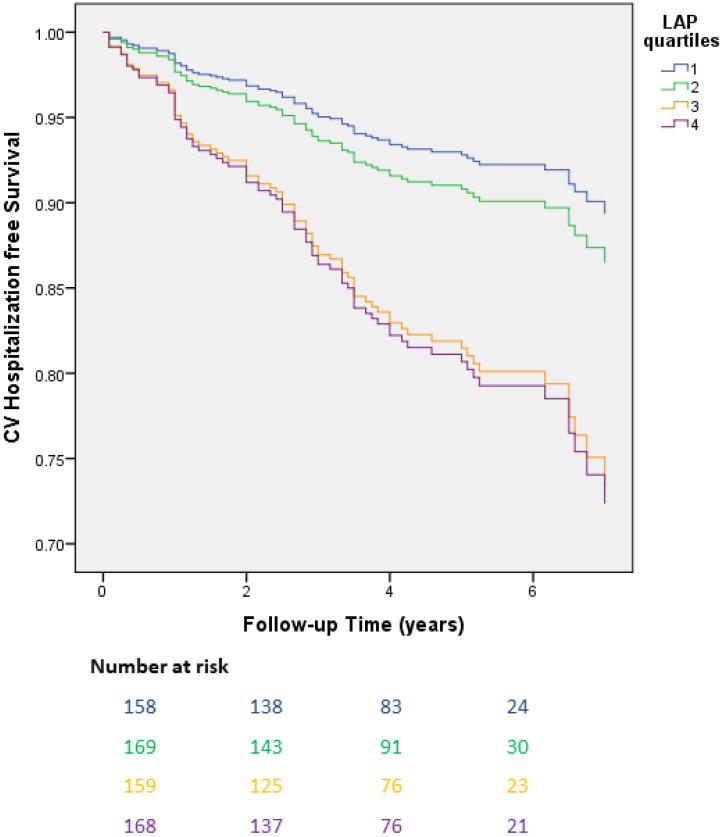
Adjusted survival curves derived from Cox regression analysis evaluating cardiovascular hospitalization by baseline lipid accumulation product (LAP) quartile. (*p*-value for LAP quartile = 0.03; interquartile comparisons: *p*-value 1st vs. 2nd = 0.48; 1st vs. 3rd = 0.01; 1st vs. 4th = 0.02).

The incidence of cardiovascular hospitalization was statistically increased in patients with higher LAP quartiles (1st quartile 8.5%, 2nd quartile 12.5%, 3rd quartile 22.2%, 4th quartile 22.6%; *p*-value for LAP quartile = 0.002), as shown Figure 1. Univariate analysis revealed that hypertension, obesity, BMI, waist circumference and LAP quartile (4th vs. 1st) are predictors for cardiovascular hospitalization. Cox regression multivariate analysis indicated that only hypertension and LAP quartile are independent predictors for cardiovascular hospitalization [hazard ratio (HR): 1.57, 95% confidence interval (CI): 1.03–2.39, *p* = 0.03; HR: 2.20, 95% CI: 1.12–4.34, *p* = 0.02, respectively] [Table 3 and Figure 2]. After adjustment for several cardiovascular risk factors, the association of LAP quartile with cardiovascular hospitalization remained robust (Supplementary Table S2).

**Figure 3:**
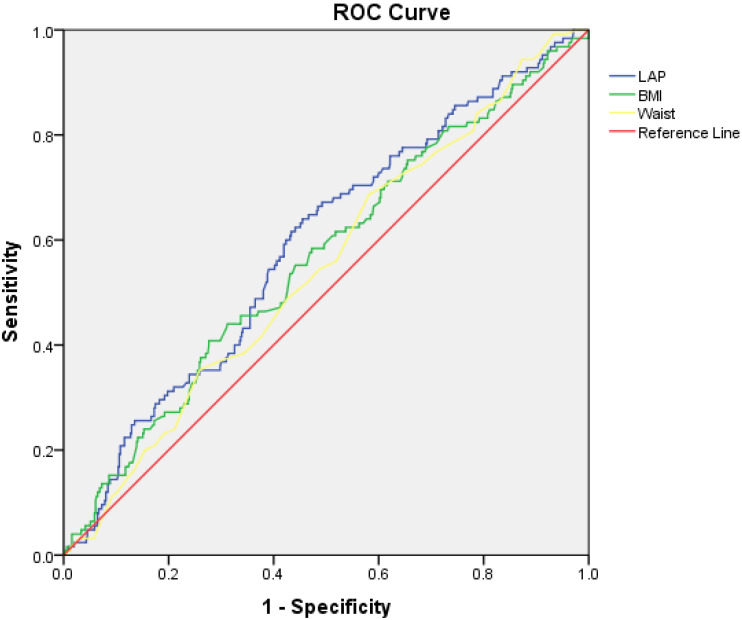
Receiver operating characteristic curves evaluating the diagnostic performance of different anthropometric indices for cardiovascular hospitalization.

ROC curve analysis revealed that LAP has better diagnostic accuracy for the prediction of cardiovascular hospitalization as opposed to BMI and waist circumference (Table 4 and Figure 3). A LAP cut-off value of 50.7 exhibited 64% sensitivity and 55% specificity for cardiovascular hospitalization.

## 4. Discussion

The main findings in our study are as follows: (1) higher LAP quartile and hypertension are independent predictors for the occurrence of cardiovascular hospitalization among patients with SIHD, (2) LAP exhibited higher diagnostic accuracy compared to other anthropometric indices (BMI, waist circumference) in relation to the study endpoint and (3) lipid goal achievement was suboptimal among SIHD patients, while a significant portion of them continue to smoke.

In our study, LAP was a better predictive marker for adverse cardiovascular events than other cardiometabolic indices such as waist circumference, BMI, triglycerides and HDL-C. Previous studies have suggested that LAP is significantly increased in metabolically unhealthy obese and non-obese individuals [21], and it is associated with abdominal obesity [22]. Furthermore, increasing LAP values have been associated with atherogenic dyslipidemia (increased apoB levels, reduced LDL and HDL particle size) and insulin resistance [23], which in turn could lead to arterial stiffness [24] and possibly coronary atherosclerosis progression [25].

ApoB-containing lipoproteins are the main drivers for atherosclerosis progression among patients with established CAD and are predictive of new MI [26,27]. We also found a significant association between LAP values and baseline apoB levels (r = 0.41, *p* < 0.0001). LAP is an emerging surrogate marker of visceral adiposity, and if further studies confirm its association with adverse cardiovascular outcomes, LAP might be proven clinically useful in monitoring atherosclerosis progression.

Although there is substantial evidence that LAP values increase cardiovascular risk in patients without established atherosclerotic cardiovascular disease [12,13,14,15], and it is associated with increased all-cause mortality in patients at high cardiovascular risk [28,29], only a few studies have addressed the impact of LAP on cardiovascular outcomes in the secondary prevention setting. In a single-center cohort of 2105 patients with ACS undergoing percutaneous coronary revascularization, LAP was an independent predictor for major cardiovascular and cerebrovascular adverse events (MACCEs) (HR 1.431, 95% CI: 1.28–1.58, *p* < 0.001) over a follow-up of 2 years and after adjustment for several cardiovascular risk factors. Moreover, a cut-off value of 57 had 55% sensitivity and 70% specificity for the development of MACCEs [30]. Although we excluded patients with a recent (within 6 months) ACS, the previously mentioned findings by Zhao et al. are in accordance with our analysis.

In a recent study, Dong et al. found that increased LAP is a robust indicator of concurrent undiagnosed comorbidities, such as coronary and carotid atherosclerosis, among hypertensive patients [31]. In our analysis, a higher LAP quartile placed patients with SIHD at increased risk for recurrent MI after adjustment for hypertension among other cardiovascular risk factors. Taken together, LAP values could be instrumental in guiding both primary and secondary cardiovascular and cerebrovascular atherosclerosis prevention among patients with hypertension.

This study is not without limitations. First, the study population was specifically selected and patients with prevalent comorbidities such as advanced age and moderate–severe chronic kidney disease were excluded. Second, we enrolled only Greek Caucasians with SIHD and thus no extrapolation should be made to other ethnicities. Third, LAP might be influenced over time by dynamic changes in waist circumference and that could have impacted on cardiovascular outcomes. Since BMI remained largely unchanged in our study, we tend to believe that waist circumference might have followed a similar trend. Finally, LLT and LDL-C target achievement should be considered in the context of the 2011 ESC/EAS Guidelines for the management of dyslipidemias, which suggested an LDL-C goal <70 mg/dL in the setting of secondary cardiovascular prevention [32].

## 5. Conclusions

LAP quartile and hypertension are predictive of adverse cardiovascular outcomes, namely non-fatal MI, non-fatal ischemic stroke and malignant ventricular arrhythmias, necessitating hospital admission among patients with SIHD. LAP exhibited higher diagnostic accuracy compared to BMI and waist circumference in relation to cardiovascular admissions and deserves further evaluation in dedicated studies.

## Figures and Tables

**Table 1:** Baseline characteristics according to the occurrence of cardiovascular hospitalization.

		CV Hospitalization		
**Parameter**	(N = 770)	Yes (N = 127)	No (N = 643)	*p*-Value
**Age (years)**	62 (55–69)	62 (55–69)	63 (55–69)	0.54
**Female gender (%)**	13	17	12	0.07
**Smoking history (%)**	79	78	80	0.71
**Hypertension (%)**	66	75	64	0.006
**Diabetes mellitus (%)**	33	40	32	0.06
**Hypercholesterolemia (%)**	55	58	54	0.63
**FHx premature CAD (%)**	38	42	37	0.07
**Prior ACS (%)**	80	81	79	0.11
**Prior revascularization** **(PCI or CABG) [%].**	77	78	75	0.52
**Waist circumference (cm)**	102 ± 12	104 ± 11	102 ± 12	0.03
**BMI (kg/m^2^)**	28 (26–32)	29 (26–32)	28 (26–31)	0.025
**Obesity; BMI > 30 Kg/m^2^ (%)**	33	42	30	0.006
**LVEF (%)**	51 (45–60)	50 (43–60)	55 (45–60)	0.63
**Total cholesterol (mg/dL)**	161 (138–191)	160 (140–188)	158 (136–189)	0.36
**LDL-C (mg/dL)**	95 (78–119)	94 (80–120)	95 (76–117)	0.33
**HDL-C (mg/dL)**	41 (35–49)	40 (34–46)	42 (36–49)	0.012
**Triglycerides (mg/dL)**	122 (89–173)	134 (92–191)	120 (89–170)	0.03
**HbA1c (%)**	6.5 (5.8–8.1)	6.5 (5.9–8.5)	6.5 (5.7–7.7)	0.29
**Lp(a) (mg/dL)**	13 (6–30)	11 (7–28)	13 (6–30)	0.85
**LAP (cm × mmol/L)**	50.6 (32.7–80.3)	60 (38.8–97.6)	47 (31.8–80)	0.002
**ASA (%)**	86	88	86	0.68
**Clopidogrel (%)**	18	16	19	0.75
**ACEi (%)**	58	59	58	0.87
**Beta-blocker (%)**	83	83	83	0.88
**Statin (%)**	89	89	89	0.97
**Ezetimibe (%)**	8	6	10	0.14
**Multivessel CAD (%)**	60.1	66.6	58.6	0.19

ASA: aspirin; ACEi: angiotensin-converting enzyme inhibitor; ACS: acute coronary syndrome; BMI: body mass index; CABG: coronary artery bypass grafting; CAD: coronary artery disease; CV: cardiovascular; FHx: family history; HbA1c: glycated hemoglobin A1; HDL-C: high-density lipoprotein cholesterol; LAP: lipid accumulation product; LDL-C: low-density lipoprotein cholesterol; Lp(a): lipoprotein(a); LVEF: left ventricular ejection fraction; Multivessel CAD: luminal diameter reduction ≥50% in at least two major coronary arteries; PCI: percutaneous coronary intervention.

**Table 2:** Follow-up data and outcomes.

		CV Hospitalization		
	Total (N = 770)	Yes (N = 127)	No (N = 643)	*p*-Value
**Primary Endpoint Components**	16.5			
**ACS (%)**	12.4	75	0	<0.001
**Non-fatal stroke (%)**	1.5	9	0	<0.001
**Ventricular arrhythmias (%)**	2.6	16	0	<0.001
**Medical treatment**				
**Antiplatelets (%)**	95	96	94	0.73
**LLT (%)**	91	90	91	0.96
**Beta-blockers (%)**	81.5	92	79	0.13
**ACEi (%)**	75.4	92	71	0.04
**Smoking persistence (%)**	21.6	27	20	0.46
**LDL-C < 70 mg/dL (%)**	17	13	17	0.12

ACEi: angiotensin-converting enzyme inhibitors; ACS: acute coronary syndrome; CV: cardiovascular; LDL-C: low-density lipoprotein cholesterol; LLT: lipid-lowering therapy.

**Table 3:** Results from the Cox proportional hazard models evaluating the development of cardiovascular hospitalization.

	Univariate Analysis			Multivariate Analysis		
Parameter	HR	95% CI	*p*-Value	HR	95% CI	*p*-Value
**LVEF (per %)**	0.98	0.97–1.00	0.07			
**Smoking persistence**	1.45	0.60–3.49	0.40			
**Hypertension**	1.68	1.16–2.41	0.005	1.57	1.03–2.39	0.03
**Diabetes mellitus**	1.35	0.98–1.88	0.06			
**Hypercholesterolemia**	1.00	0.99–1.00	0.58			
**Lipoprotein(a) (per mg/dL)**	1.00	0.99–1.00	0.86			
**Triglycerides (per mg/dL)**	1.00	1.00–1.00	0.12			
**HDL-C (per mg/dL)**	0.96	0.92–1.10	0.09			
**Baseline BMI (per kg/m^2^)**	1.06	1.02–1.09	0.001	1.01	0.95–1.07	0.65
**BMI at follow-up** **(per kg/m^2^)**	1.01	0.97–1.06	0.45			
**Waist (per cm)**	1.01	1.00–1.03	0.01	0.99	0.96–1.01	0.48
**Obese (BMI > 30 kg/m^2^)**	1.59	1.14–2.2	0.05	1.27	0.85–1.88	0.23
**LAP quartile (4th vs. 1st)**	2.36	1.37–4.04	0.02	2.20	1.12–4.34	0.02
**Age (per year)**	0.99	0.98–1.01	0.90			
**Female gender**	0.71	0.47–1.07	0.10			

BMI: body mass index; HDL-C: high-density lipoprotein cholesterol; HR: hazard ratio; LAP: lipid accumulation product; LVEF: left ventricular systolic function; 95% CI: 95% confidence interval.

**Table 4:** Diagnostic performance of different anthropomorphic indices for cardiovascular hospitalization.

Variable	Area under the Curve	95% Confidence Intervals	*p*-Value
**LAP**	0.59	0.53–0.64	0.002
**BMI**	0.56	0.50–0.62	0.026
**Waist circumference**	0.54	0.49–0.60	0.09

LAP: lipid accumulation product, BMI: body mass index.

## Data Availability

Data are available upon reasonable request.

## Supplementary Materials

The following supporting information can be downloaded at https://www.mdpi.com/article/10.3390/jcdd11100316/s1, Table S1: Association of LAP with baseline cardiometabolic parameters, Table S2: Adjusted survival analysis models, Figure S1: Correlation between lipid accumulation product (LAP) and Apolipoprotein B.
