# Supplementary material for: Lipid Accumulation Product Is Predictive of Cardiovascular Hospitalizations among Patients with Stable Ischemic Heart Disease: Long-Term Follow-Up of the LAERTES Study
Source: J Cardiovasc Dev Dis. 2024 Oct 10;11(10):316. doi: 10.3390/jcdd11100316 (PMC11508490; doi:10.3390/jcdd11100316)
Supplement: Supplementary file 1 [file jcdd-11-00316-s001.zip › jcdd-3226021-supplementary.pdf]

**Supplementary Table S1**: Association of LAP with baseline cardiometabolic parameters		
	LAP (Spearman's rho)	**p-value**
BMI	0.55	<0.0001
Waist circumference	0.67	<0.0001
SBP	0.20	<0.0001
DBP	0.18	<0.0001
Fasting Glucose	0.23	<0.0001
HbA1c	0.14	0.04
Total Cholesterol	0.29	<0.0001
LDL-C	0.16	<0.0001
HDL-C	-0.27	<0.0001
Triglycerides	0.83	<0.0001
Apolipoprotein B	0.41	<0.0001

LAP: lipid accumulation product, BMI: body mass index, SBP: systolic blood pressure, DBP: diastolic blood pressure, HbA1c: glycated hemoglobin A1, LDL-C: low-density lipoprotein-cholesterol, HDL-C: high-density lipoprotein-cholesterol

Supplementary Table S2: Adjusted survival analysis models

**Parameter**	**Hazard ratio**	**95% Confidence Intervals**	**p-value**
LAP quartiles (4th vs 1st)*	2.35	1.37-4.03	0.002
LAP quartiles (4th vs 1st)†	1.99	1.13-3.49	0.02
LAP quartiles (4th vs 1st)‡	2.09	1.07-4.07	0.03

*Model 1 adjusted for age and gender
†Model 2 adjusted for age, gender, hypertension, diabetes mellitus, dyslipidemia and smoking
‡Model 3 adjusted for age, gender, hypertension, diabetes mellitus, dyslipidemia, smoking, previous myocardial infarction, ejection fraction and multivessel coronary artery disease

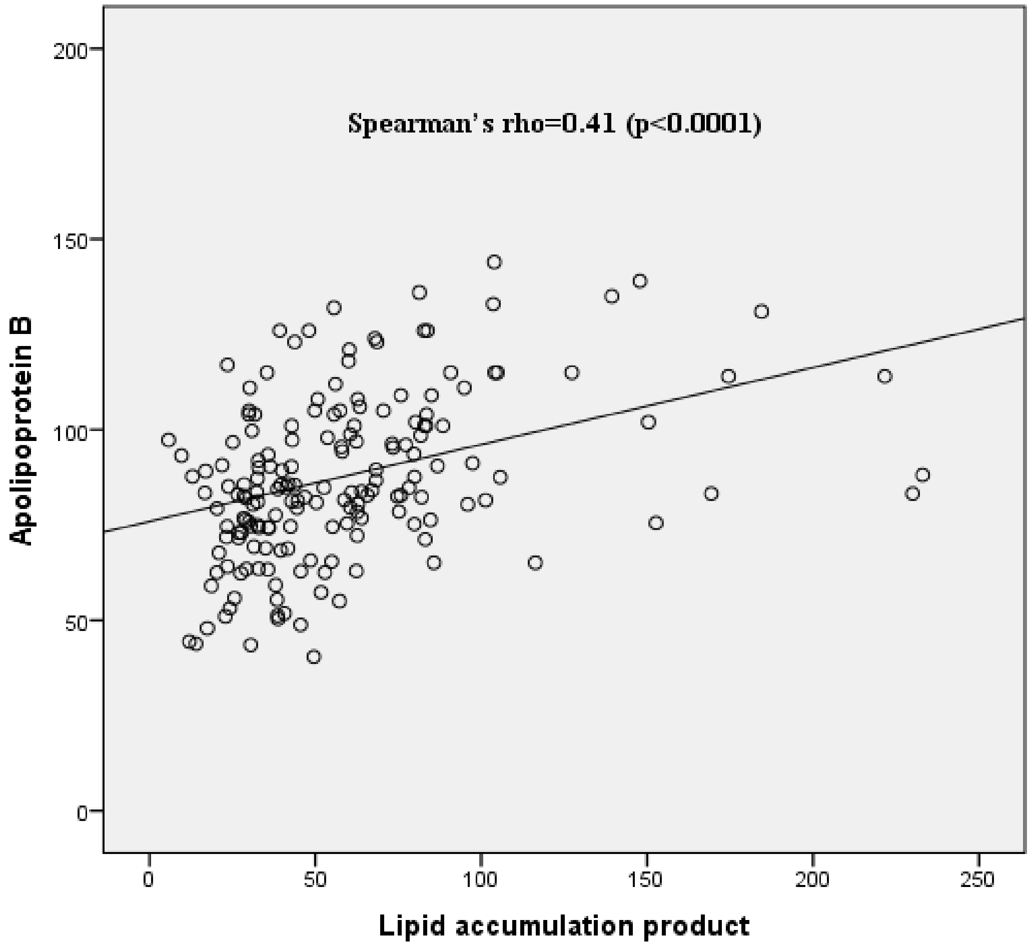

Supplementary Figure S1: Correlation between Lipid Accumulation Product (LAP) and Apolipoprotein B.
